# The Value of EphB2 Receptor and Cognate Ephrin Ligands in Prognostic and Predictive Assessments of Human Breast Cancer

**DOI:** 10.3390/ijms22158098

**Published:** 2021-07-28

**Authors:** Abdul Shukkur Ebrahim, Zeyad Hailat, Sudeshna Bandyopadhyay, Daniel Neill, Mustapha Kandouz

**Affiliations:** 1 Department of Ophthalmology, Visual & Anatomical Sciences, Wayne State University School of Medicine, Detroit, MI 48201, USA; eabdulsh@med.wayne.edu; 2 Department of Computer Science, Wayne State University, Detroit, MI 48201, USA; zmhailat@gmail.com; 3 Department of Pathology, Wayne State University School of Medicine, Detroit, MI 48201, USA; sbandyop@med.wayne.edu (S.B.); daniel.neill@wayne.edu (D.N.); 4 Karmanos Cancer Institute, Wayne State University, Detroit, MI 48201, USA

**Keywords:** intercellular communication, EphB2, ephrin, breast cancer, prognosis

## Abstract

Cell–cell communication proteins Eph and ephrin constitute the largest family of receptor tyrosine kinases (RTKs). They are distinguished by the fact that both receptors and ligands are membrane-bound, and both can drive intracellular signaling in their respective cells. Ever since these RTKs have been found to be involved in cancer development, strategies to target them therapeutically have been actively pursued. However, before this goal can be rationally achieved, the contributions of either Eph receptors or their ephrin ligands to cancer development and progression should be scrutinized in depth. To assess the clinical pertinence of this concern, we performed a systematic review and meta-analysis of the prognostic/predictive value of EphB2 and its multiple cognate ephrin ligands in breast cancer. We found that EphB2 has prognostic value, as indicated by the association of higher *EphB2* expression levels with lower distant metastasis-free survival (DMFS), and the association of lower *EphB2* expression levels with poorer relapse-free survival (RFS). We also found that higher *EphB2* expression could be a prognostic factor for distant metastasis, specifically in the luminal subtypes of breast cancer. *EFNB2* showed a marked correlation between higher expression levels and shorter DMFS. *EFNA5* or *EFNB1* overexpression is correlated with longer RFS. Increased *EFNB1* expression is correlated with longer OS in lymph node (LN)-negative patients and the luminal B subtype. Higher levels of *EFNB2* or *EFNA5* are significantly correlated with shorter RFS, regardless of LN status. However, while this correlation with shorter RFS is true for *EFNB2* in all subtypes except basal, it is also true for *EFNA5* in all subtypes except HER2+. The analysis also points to possible predictive value for *EphB2*. In systemically treated patients who have undergone either endocrine therapy or chemotherapy, we found that higher expression of *EphB2* is correlated with better rates of RFS. Bearing in mind the limitations inherent to any mRNA-based profiling method, we complemented our analysis with an immunohistochemical assessment of expression levels of both the EphB2 receptor and cognate ephrin ligands. We found that the latter are significantly more expressed in cancers than in normal tissues, and even more so in invasive and metastatic samples than in ductal carcinoma in situ (DCIS). Finally, in an in vitro cellular model of breast cancer progression, based on H-Ras-transformation of the MCF10A benign mammary cell line, we observed dramatic increases in the mRNA expression of *EphB2* receptor and *EFNB1* and *EFNB2* ligands in transformed and invasive cells in comparison with their benign counterparts. Taken together, these data show the clinical validity of a model whereby EphB2, along with its cognate ephrin ligands, have dual anti- and pro-tumor progression effects. In so doing, they reinforce the necessity of further biological investigations into Ephs and ephrins, prior to using them in targeted therapies.

## 1. Introduction

Eph receptors and their cognate ephrin ligands belong to a family that constitutes the largest group of receptor tyrosine kinases (RTK). They are responsible for countless essential biological functions, including the development of the central nervous system, embryonic tissue morphogenesis, and angiogenesis [1,2,3,4,5,6,7,8,9,10,11,12,13,14]. These proteins are clustered into two classes, A-type (EphA) and B-type (EphB) receptors, and there are A-type (ephrin-A) and B-type (ephrin-B) ligands. With only few exceptions, EphA receptors bind exclusively to ephrin-As, and EphB receptors bind almost exclusively to ephrin-Bs. Eph receptors and ephrin ligands operate via direct cell–cell contact. In fact, a distinctive feature is that they are both membrane-bound. Ephrin-As are tethered to the cell surface by a glycosyl-phosphatidylinositol (GPI)-anchor, and ephrin-Bs are bound to the membranes by a single transmembrane domain [15,16]. Therefore, both require direct cell–cell contact to mediate their respective functions, and upon activation, a bi-directional signaling event takes place: both through the receptor (called forward signaling) and through the ligand (called reverse signaling). This configuration has a significant functional impact on neighboring cells. Different outcomes could be reached depending on aberrant expression and/or aberrant functioning of one or both partners in this interaction. There is strong evidence that this concept underlies the finding that disruption of a single Eph or ephrin can cause multiple and sometimes paradoxical results. This is reminiscent of what is encountered in cancer [17], whereby Ephs and ephrins appear to behave both as oncogenes and as tumor suppressors [18]. An example of a particular focus in our laboratory is investigating the roles of EphB2 and ephrin ligands in breast cancer [19,20,21,22]. We have shown that EphB2 expression promotes three different homeostatic processes: autophagy, apoptosis, and surprisingly, invasion [23]. In line with this finding, our research shows that while EphB2 is expressed in benign tissues, it is highly upregulated in breast cancer, particularly in invasive and metastatic carcinomas [23].

Although our previous findings shed light on the role of EphB2 in breast cancer, they raise questions about the possibility of dual or even apparently contradictory functions. One possible element of understanding this ambiguity is the co-expression of cognate ephrin ligands. Unfortunately, data regarding the role of cognate EphB2 ligands specifically in breast cancer is scarce. Ephrin-B2 expression was shown to have negative effects on proliferation and motility and to be associated with longer patient survival in breast cancer [24]. Its co-expression was shown to oppose the pro-oncogenic function of EphB4, another Eph receptor of importance in breast cancer [25]. Higher expression of ephrin-B1 appears to be related to breast cancer metastasis and confers a poor prognosis [26]. Using a proteomic approach, it has been found to be one of the proteins associated with metastatic tropism to the brain, at least of the MDA-MB-231 cell line [27]. To the best of our knowledge, there are almost no data on ephrin-B3 and ephrin-A5 in breast cancer.

In the present study, we assessed the prognostic significance of expression of the EphB2 receptor and ephrin ligands in breast cancer, as a first exploration of the clinical validity of our hypothesis that ephrin ligands determine the pro- and anti-cancer roles of EphB2.

## 2. Results

### 2.1. EphB2 Expression Is an Adverse Prognostic Factor for Distant Metastases and a Positive Predictive Factor for Post-Therapy Relapse

**Figure 1:**
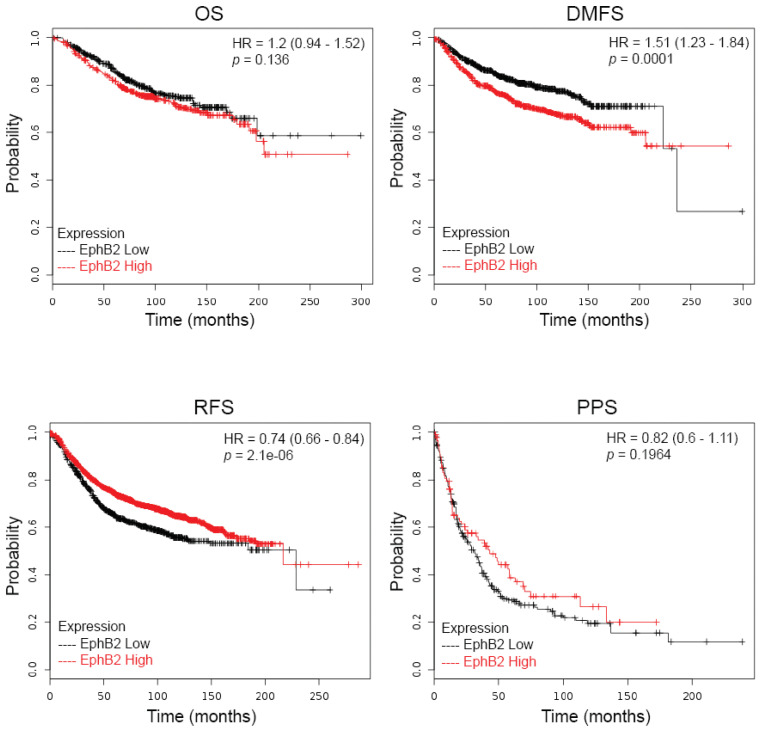
Prognostic value of EphB2 expression in breast cancer. Kaplan–Meier curves were generated using four types of breast cancer patient survival data from www.kmplot.com: overall survival (OS), relapse-free survival (RFS), distant metastasis-free survival (DMFS), and post-progression survival (PPS). Survival of two groups of patients, with low versus high EphB2 mRNA expression levels, was compared, and both hazard ratios (HR) with 95% confidence intervals and log-rank *p* values were calculated.

To assess the possible contributions of *EphB2* to the prognosis of cancer patients, we determined the relationship of *EphB2* expression with survival, using the KMPLOT clinical microarray database, designed for meta-analysis-based biomarker assessment [28], and which includes 4142 patients with a follow-up of 69/40/49/33 months. In this database, out of four different *EphB2* probe sets, only probe 209588_at (RefSeq Transcript ID: NM_004442, NM_017449) was used in this analysis, and the remaining probe sets were deemed of less satisfactory quality by the database criteria and thus excluded. To this end, quality control was based on a scoring method established to assess specificity, coverage, and resistance to degradation [29]. However, no data set was disconfirmed, whether it impacted significance or not; rather, all findings were added and analyzed together. Four different patient survival assays are available: overall survival (OS), relapse-free survival (RFS), distant metastasis-free survival (DMFS), and post-progression survival (PPS). To summarize the results, we found no significant correlation between levels of *EphB2* expression and OS or PPS, but we found that higher *EphB2* levels predict poorer DMFS (*p* value = 0.0001) (Figure 1), and lower *EphB2* levels correlate with poorer RFS (*p* value = 2.1 × 10^−6^ (Figure 1).

**Figure 2:**
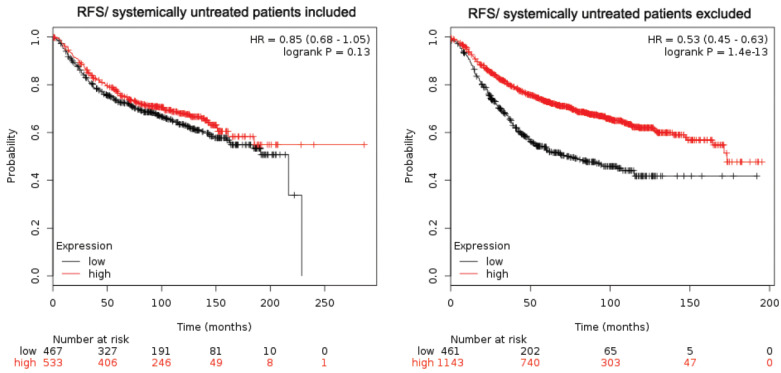
The confounding effect of systemic therapy on the prognostic value of EphB2 in breast cancer. Kaplan–Meier curves were generated using relapse-free survival (RFS) from the survival data of www.kmplot.com, depending on inclusion or not of systemically treated patients. For each analysis, survival of two groups of patients, with low versus high EphB2 mRNA expression levels, was compared, and both hazard ratios (HR) with 95% confidence intervals and log-rank *p* values were calculated.

An in-depth look at RFS showed a few interesting findings. When the RFS analysis was performed only on systemically treated patients (cohort analysis restricted to “systemically untreated patients excluded,” meaning that only patients who underwent either endocrine therapy or chemotherapy were included), higher expression of *EphB2* was correlated with better survival (*p* value = 1.4 × 10^−13^) (Figure 2). In contrast, when the systemically untreated patients were included (cohort analysis restricted to “systemically untreated patients included,” meaning that patients were included regardless of whether they had received systemic therapy or not), there was no significant correlation of *EphB2* expression (*p* value = 0.13; Figure 2) with RFS. This result suggests that systemic therapy might be a confounding factor in assessing the prognostic value of *EphB2*.

### 2.2. The Prognostic Value of EphB2 Is Stronger in the Luminal Subtypes of Breast Cancer

An important insight into the heterogeneous nature of breast cancer has been provided by the molecular classification of this disease into four major molecular subtypes. These subtypes have been categorized as luminal A, luminal B, triple-negative breast cancer (TNBC)/Basal-like, and HER2-enriched [30,31,32]. Each group demonstrates clinical behavior and responses to therapy associated with a specific gene expression pattern, the presence of absence of a hormone receptor (estrogen-receptor ER and/or progesterone-receptor PR positive) and HER2 enrichment. The luminal A subtype is associated with better prognosis. The HER2 and the basal subtypes tend to have worse clinical outcomes [31,32,33].

We were interested in learning whether expression of *EphB2* fits into these established categories. To that end, we assessed different clinical outcomes according to *EphB2* expression in each subtype of breast cancer. We discovered that the luminal subtypes showed significant correlations between higher levels of expression of *EphB2* and poor DMFS (*p* value = 0.026 for luminal A; *p* value = 0.0014 for luminal B) (Table 1). Although the examined group of cases categorized as basal or HER2+ subtypes was limited, we did not find notable differences in survival rates between patients with high vs. low *EphB2* expression.

When OS was used as a clinical endpoint, none of the differences reached significance among the different subtypes.

When RFS was assessed, luminal A, luminal B, and basal subtypes showed modest correlations between lower *EphB2* expression and poorer RFS (Table 1). This reflects the trend observed for all patients, that is, regardless of subtype (Figure 1).

**Figure 3:**
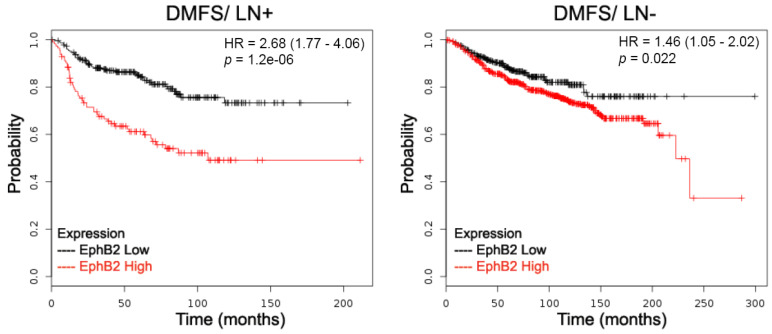
Prognostic value of EphB2 expression in distant metastases. EphB2 expression levels were correlated with distant metastasis-free survival (DMFS) and lymph node status. Survival of two groups of patients, with low versus high EphB2 mRNA expression levels, was compared: patients with positive lymph nodes (LN+) versus patients with negative lymph nodes (LN-). Both hazard ratios (HR) with 95% confidence intervals and log-rank *p* values were calculated.

As the focus of our investigation is the prognostic value of *EphB2* expression, we expanded our analysis to include both subtype and lymph node status. When lymph node status was included, there was a striking correlation between higher levels of *EphB2* expression and shorter DMFS in node-positive carcinoma patients (Table 1, Figure 3). This reinforces the association that was observed between higher EphB2 levels and metastasis [23]. We also observed correlations between higher levels of *EphB2* expression and shorter DMFS for luminal type A and B patients, but not for basal and HER2-positive patients (Table 1, Figure 3). The finding that high *EphB2* levels associate with shorter DMFS suggests that *EphB2* could be a predictor of metastatic progression, specifically in luminal types of breast cancer. All other correlations were either not significant or relied on too few patients (KM Plotter).

### 2.3. Correlations between Expression Levels of EphB2 Ligands and Patient Survival

As we previously mentioned, one consequence of the membrane-bound nature of Eph-family receptors and ephrin ligands is the need for the communicating cells to be in direct contact to initiate signaling. The impact of *EphB2* gene expression on breast cancer is likely to be influenced by the co-expression of the genes that encode ephrin (EFN) ligands. Among the ephrins, EphB2 is known to bind ephrin-B1 (encoding gene: *EFNB1*), ephrin-B2 (encoding gene: *EFNB2*), ephrin-B3 (encoding gene: *EFNB3*), and ephrin-A5 (encoding gene: *EFNA5*) [15,16,34,35,36,37]. Therefore, we were interested to know whether the expression of one or more of these ligands could be correlated with breast carcinoma behavior and thus provide more detailed prognostic information. We performed a comprehensive evaluation of the correlations between the expression of EphB2 ligands and survival rates, regarding various factors (i.e., lymph node status and subtypes). The analysis, summarized in Table 1, shows several—at times intriguing—results.

When DMFS was used as a clinical endpoint, two of the four ligands showed a correlation between increased expression and shorter survival time. *EFNB2* (probe set 202668_at, RefSeq Transcript ID: NM_004093) showed a marked correlation between higher expression levels and shorter survival (HR = 1.43; *p* = 0.00058). *EFNB1* (probe set 202711_at, RefSeq Transcript ID: NM_004429) showed a similar trend, although at lower significance (HR = 1.25; *p* = 0.038). Neither *EFNA5* (214036_at, RefSeq Transcript ID: NM_001962) nor *EFNB3* (probe set 205031_at, RefSeq Transcript ID NM_001406) showed significant differences (Table 1).

When RFS was used as the clinical endpoint, *EFNA5* and *EFNB1* showed the same prognostic features of *EphB2*: overexpression of either one was correlated with longer RFS in the whole patient group.

When OS was the clinical endpoint, increased *EFNB1* expression was correlated with longer survival in lymph node-negative patients and the luminal B subtype.

Higher levels of *EFNB2* were significantly correlated with shorter survival (RFS) in all samples regardless of LN status, and in all subtypes except basal. Higher levels of *EFNA5* were also significantly correlated with shorter survival (RFS) in all samples regardless of LN status and in all subtypes except HER2+.

### 2.4. Co-Expression of EphB2 and Cognate Ligands Is Correlated with Breast Cancer Progression

**Figure 4:**
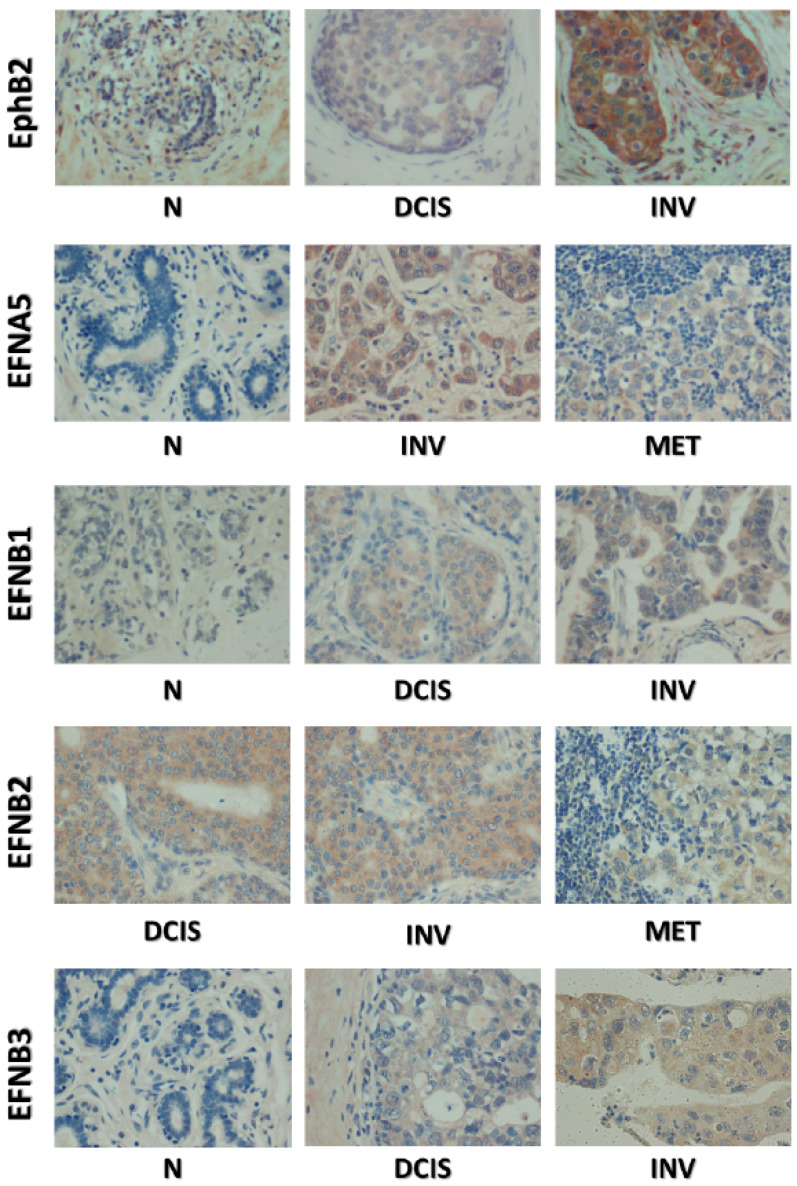
Immunohistochemical staining of EphB2 and cognate ephrin ligands in human breast clinical specimens. Representative microphotographs were taken at ×200 magnification, and include normal samples (N), ductal carcinomas in situ (DCIS), invasive carcinomas (INV), and metastases (MET).

As stated above, EphB2 is known to bind ephrins B1, B2, B3, and A5. We examined how expression of both receptor and ligands correlates with disease progression in breast cancer. In a previous work, we found that although EphB2 is expressed in normal and benign tissues, its levels in these tissues are lower than in cancer tissues (Table 2, Figure 4) [23]. Indeed, to aid in assessing the levels of EphB2 expression, we developed a scoring system with “non-cancer” tissues used as the baseline reference value (defined as 0). Using this scoring system, the median scores were intraductal (80, *p* < 0.0001), invasive ductal (35, *p* < 0.0001), and metastasis (90, *p* < 0.0001) [23]. When we extended this analysis to the ephrin ligand proteins (B1, B2, B3, and A5), using a similar scoring system, we found that they all are significantly more expressed in cancers than in normal tissues (Table 2, Figure 4). Despite this general qualitative increase in expression, there are quantitative differences: ephrin-B1, B2, and A5 are far more highly expressed in invasive and metastatic samples than in DCIS, and the frequency of ephrin-B3 expression in all cancers seems to be lower than the other ligands.

**Figure 5:**
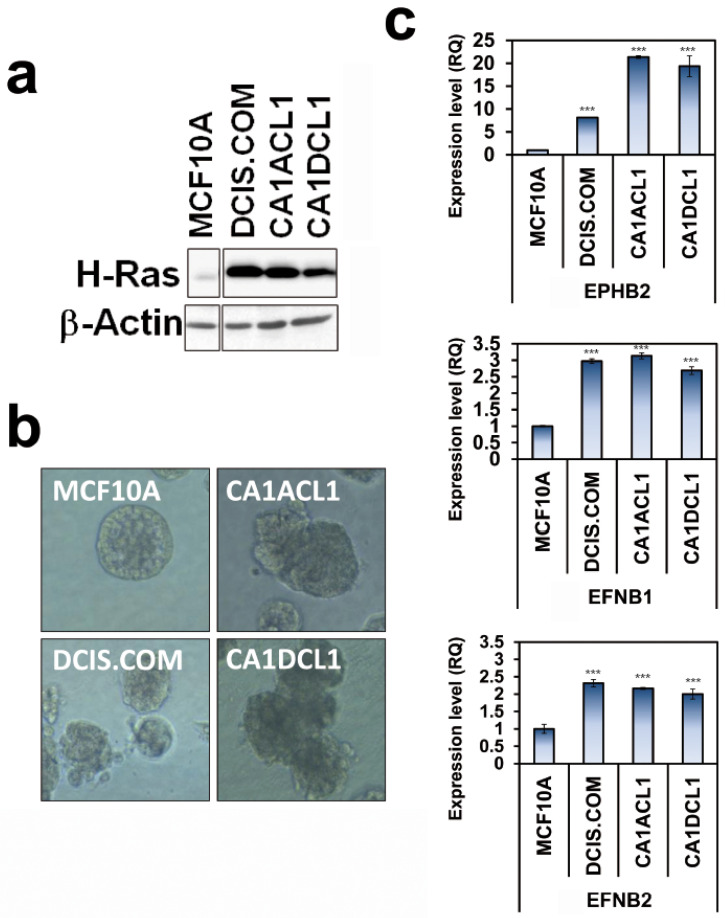
Expression of EphB2 and ligands EFNB1 and EFNB2 in an MCF10A-derived cellular model of breast cancer progression. (**a**) An immunoblot of H-Ras-transformed MCF10A-derived cells. (**b**) Phase contrast and immuno-fluorescence images illustrating the morphologies of normal MCF10A, derived DCIS.com, and invasive CA1acl1 and CA1dcl1 grown in 3D (Nuclear blue staining: DAPI, membrane green staining: β4 integrin). After 10 days of culturing, MCF10A cells form polarized spherical acini that have hollow lumens. Transformed cell lines form complex, non-polarized, and dense masses. Magnification: 40×. (**c**) Real-time RT-PCR of expression of *EphB2* and ligands in MCF10A-derived cells. RQ: Relative quantity. *** *p* < 0.005. *t*-test. GraphPad Prism5.

A similar trend towards co-expression of *EphB2* and cognate ligands along cancer progression was also observed in cell lines. Indeed, we examined cell lines derived from MCF10A cells, a well-established cellular model of spontaneously immortalized non-transformed and non-tumorigenic human mammary epithelial cells [16], which have been engineered to express Harvey-*ras* (H-*ras*) (Figure 5a). This model was derived to precisely recapitulate the progressive alterations associated with the temporal development of human breast carcinomas [38,39,40,41,42]. The model included, in addition to parental MCF10A cells, ductal carcinoma in situ (DCIS.com) and invasive (CA1ACL1 and CA1DCL1) cell variants (Figure 5a,b). DCIS.com cells are not only able to develop DCIS-like lesions and to generate both epithelial luminal and myoepithelial cells in vivo; they also undergo spontaneous progression to invasive cancer. When grown in 3D cultures, the transformed MCF10A-derived cells show morphogenic structures different from the typical round MCF10A structures and reminiscent of transformed clinical features (Figure 5b). We collected cells grown in 3D and examined the mRNA expression levels of *EphB2* and its major cognate ligands *EFNB1* and *EFNB2* via real-time qRT-PCR. The results show dramatic increases in the expression of *EphB2* receptor and *EFNB1* and *EFNB2* ligands in transformed and invasive cells in comparison with benign MCF10A cells (Figure 5c). This result confirms the data we observed in clinical specimens, whether at the mRNA (KM plotter) or protein (IHC) levels.

## 3. Discussion

During previous studies, we and others encountered multiple functions for Eph and ephrin proteins. Our earlier work demonstrated that EphB2 expression suppresses the growth of human breast cancer cells both in vitro and in vivo [23]. We identified a regulatory mechanism that involves both autophagy and apoptosis. However, paradoxically, while EphB2 expression induces both autophagy and apoptosis, we also found that it promotes cell invasiveness [23]. This made us reconsider our initial hypothesis that EphB2 might be a bona fide tumor suppressor, and that it might rather possess a dual context-dependent function, mainly modulated by co-expression of cognate ephrin ligands. Indeed, the functions of EphB2 are likely to be regulated by the co-expression and mutual activation by and of its ephrin ligands. This role of the ligands is further emphasized by the fact that in the Eph/ephrin family, the membrane-bound ligands behave as receptors that elicit their own so-called reverse signaling. The present work is a step further in assessing the accuracy of this hypothesis, using clinical materials. Here we analyzed the clinical significance of concomitant expression of *EphB2* and its cognate ephrin ligands in the context of breast cancer progression. We performed a comprehensive evaluation of the correlations among *EphB2* and *EFN* expression and survival rates, with regard to various clinicopathological factors.

Our present analysis shows an association between *EphB2*, *EFNB2*, or *EFNB1* overexpression and shorter DMFS on one hand, and between *EphB2*, *EFNA5*, or *EFNB1* and longer RFS on the other hand. From these data, along with in vitro and in vivo results [23], we conclude that *EphB2* is moderately expressed and might behave as a tumor suppressor in normal and benign mammary tissues, despite being overexpressed in later stages, when it is associated with invasiveness and metastasis. The present data also support a role of concomitant increases in the expression of the EphB2 receptor and its ephrin ligands during breast cancer progression and metastasis. However, this co-expression of receptor and ligands was observed also in in situ (DCIS) cases as well, as assessed by immunohistochemistry. The same finding was observed in the MCF10A-derived model of breast cancer progression, whereby both the invasive (CA1ACL1 and CA1DCL1) and the DCIS.com cell variants co-expressed higher levels of *EphB2*, *EFNB1*, and *EFNB2* than the benign parental MCF10A cell line. These results suggest that downregulating the expression of *EphB2* and ligands’ mRNAs is an early event in breast cancer progression, which is maintained and might have specific functions that vary during progression.

Regarding the clinical significance of our analysis, we found that EphB2 expression is an adverse prognostic factor for distant metastases and a positive predictive factor for relapse. It is thus possible that, although we have uncovered different EphB2 functions using in vitro and in vivo models, the actual coordination of these functions and the dominance of one function over the others might depend on other clinical parameters. In other words, increases in EphB2 levels might result in distinct functions depending on the cellular and molecular context and patient therapeutic history, rather than being of an overall fixed and linear pattern throughout tumor progression. First, systemic therapy might be a confounding factor in assessing the prognostic value of *EphB2*. It is possible that this is in line with the paradoxical impact of chemotherapy, which ensures therapeutic efficacy regarding the primary tumor, despite eventually favoring survival and dissemination of metastatic cancer cells [43,44]. A hypothesis that merits further investigation is that loss of *EphB2* expression could be associated with better growth of cancer cells (hence the correlation between lower *EphB2* levels and poorer RFS). Another is that overexpression of *EphB2* in metastasizing cells favors their survival and growth (hence the correlation between higher *EphB2* levels and poorer DMFS). Second, as our analysis shows, the prognostic value of *EphB2* is strong in the luminal subtypes of breast cancer but not in basal or HER2-positive subtypes, all of which are known to have different biochemical and cellular characteristics. An interpretation of this correlation would be highly speculative at this point in our investigation. It is unlikely that this is due to the ER/PR hormonal status in luminal types, since analyzing the data in ER/PR-positive vs. ER/PR-negative patients did not reveal significant differences (KM Plotter). However, these data have a potentially important impact. Indeed, luminal types are highly heterogeneous and are characterized by different risks of relapse. The fact that high *EphB2* levels associate with shorter DMFS suggests a new line of investigation, whereby *EphB2* could be viewed as a predictor of metastatic progression, specifically in luminal types of breast cancer.

As mentioned above, we intended this evaluation to not only assess the value of these genes as clinical markers of survival outcomes, but to also refine our understanding of their biological functions in breast cancer. To what extent do these data advance our understanding of the function of EphB2 and ligands at the molecular level? Our previous work shows that, like other tumor suppressors, EphB2 has pro-apoptotic functions. This data came along with a paradox—the fact that EphB2 is overexpressed in cancer tissues [23] and that it also induces cancer cell invasion [45]. Our earlier finding provided a piece of explanation: inhibiting autophagy can significantly block EphB2-induced apoptosis [45], thereby suggesting that autophagy might mediate EphB2-driven apoptosis in mammary cells. This hypothesis is supported by the fact that autophagy is connected with both pro-survival and pro-apoptotic mechanisms [46,47,48,49,50,51,52,53,54]. In addition, we also hypothesized the importance of ligands for favoring one function of EphB2 over another. With this in mind, the finding that EphB2/ephrin co-expression is observed in DCIS could be of specific biological significance. For example, it might be that in the DCIS tumors, which express EphB2, the pro-autophagic function of this receptor is dominant, thereby favoring a pro-survival and pro-oncogenic mechanism. In fact, it was previously shown that autophagy is required for the survival of abnormal precursor cells that pre-exist in DCIS [55]. We are in the process of examining the impact of ephrin binding on the pro-autophagic function of EphB2 in breast cancer. Interestingly, it has been shown that EphB2-ephrin-B1 interaction regulates autophagy in colonic epithelial cells [56]. It is therefore plausible that in DCIS, the interaction of EphB2 with cognate ephrin ligands could control its pro-autophagic function in mammary cells and subsequently favor its pro-apoptotic role.

For more advanced cancer stages, conflicting data regarding the impacts of EphB2 and ligands on invasiveness and metastatic progression have been reported, although none of them for breast cancer. For instance, a trend toward decreased EphB2 expression was observed in metastatic lesions of colorectal cancers (CRC), but no significant association was observed between EphB2 expression and advanced tumor grade in this study [57]. Additionally, CRC patients with lower EphB2 expression were found to have more advanced tumor stages, poor differentiation, poor overall survival, and poor disease-free survival [58]. Another study suggested that low expression of EphB2 is correlated with CRC metastasis to the liver [49]. Overexpression of EphB2 inhibited colon cancer migration [58]. On the other hand, it has also been reported that migrating glioblastoma (GBM) cells express high levels of EphB2 in vitro and in vivo [59], and that EphB2 overexpression in glioma cells results in increased cell invasion [60]. Using a model of GBM neurosphere formation, it was shown that EphB2 expression stimulates GBM cell migration and invasion [61]. Here again, information is scarce regarding the modulation of the role of EphB2 in migration and invasion by cognate ephrin ligands. Our assessment of the clinical material suggests that these ligands could favor the pro-invasive behavior of EphB2. EphB2-ephrin-B1 induces the invasiveness of pancreatic cancer cells [62]. Similarly, EphB2, by interacting with either ephrin-B1 [59] or ephrin-B3 [63], stimulates invasion in glioma cells. Ephrin-B1 was also shown to be important for EphB2-induced invasion in at least one breast cancer cell line [64]. However, in this latter case, the authors assessed the ephrin-B1-initiated reverse signaling rather than the EphB2-initiated forward signaling.

Since the current study was focused on the prognostic and predictive value of EphB2 and ligands, we did not address molecular mechanisms at play, and many essential questions remain unanswered at this point. Importantly, is there a global impact of the co-expression of most or all ligands on the behavior of the EphB2 receptor, or are there specificities when ligands are expressed individually? We know, for example, that at least structurally, the interaction of EphB2 with ephrin-A5 is distinct from that with ephrin-B2 [16]. In another respect, it should be noted that the current study is related to homotypic communication between mammary epithelial cells, as we assessed co-expression of EphB2 and ephrin ligands in the same cell types. However, there is another important but distinct aspect to the EphB2/ephrin-mediated role in breast cancer, the heterotypic communication between mammary cells and stromal/endothelial cells. For instance, EphB2 proteins carried by extracellular vesicles derived from head and neck squamous cell carcinoma (HNSCC) show pro-angiogenic effects, possibly by stimulating ephrin-B reverse signaling on neighboring endothelial cells [65]. These questions await further investigation.

In conclusion, the present correlative data, in conjunction with our previous in vitro and in vivo data [23], led to a model whereby in a normal context, EphB2-mediated autophagy triggers pro-apoptotic signals, resulting in tumor growth inhibition. However, in a cancer-initiation context, a blockade of apoptosis occurs, possibly driven by co-expression of and mutual co-activation by cognate ephrin ligands. This event would favor the pro-survival function of autophagy, thereby allowing the emergence of the pro-invasive function of EphB2.

The translational impact of this study is significant. There are few data regarding the role of many Ephs/ephrins in breast cancer. These were mainly reported for the A-type EphA2 and the B-type EphB4 and EphB6 [66,67,68,69,70,71,72,73,74,75,76,77,78,79]. As a result of research done in the previous decade, much work is being done to target Eph receptors in multiple malignancies, including breast cancer [80,81]. However, given the contradictory nature of the data on the biological impact of Eph receptors on the growth of various tumors, i.e., pro-tumor progression effects in certain conditions and anti-tumor effects in others, this therapeutic quest requires more detailed biological investigations before an application of Eph targeting can be reliably developed. In other words, it is critical to understand the biology of these molecules if they are to be targeted therapeutically.

## 4. Materials and Methods

### 4.1. Kaplan–Meier Plot Analysis

Correlations of gene expression levels and clinical parameters were obtained using an online database, the KM Plotter (https://kmplot.com/analysis/), a microarray database designed for meta-analysis-based biomarker assessment [28], and which includes 4142 patients with a follow-up of 69/40/49/33 months. The database allowed us to determine gene transcript expression, hazard ratios, confidence intervals, and log-rank *p* values for EphB2 receptor and cognate ligands-encoding genes, in relation to overall survival (OS), relapse-free survival (RFS), distant metastasis-free survival (DMFS), or post-progression survival (PPS), in breast cancer clinical specimens. Kaplan–Meier survival plots, hazard ratios (HR), 95% confidence intervals (CI), and log rank *p* were obtained; and a *p* value of <0.05 was considered statistically significant. Samples were automatically grouped according to the median (or upper or lower quartile) expression of the queried gene, and then the two groups were compared via a Kaplan–Meier plot. Auto-selected best cutoff was used for the expression analysis. As per the KM plotter, “all possible cutoff values between the lower and the upper quartiles are computed, and the best performing threshold was used as a cutoff”. Data in this database correspond to patients that are unidentifiable by the authors.

### 4.2. Tissue Microarray and Immunohistochemistry

We analyzed the protein expression levels of the EphB2 receptor and ephrin ligands in a panel of human benign and cancer breast specimens using a commercially available TMA (Breast Cancer Progression Tissue Array BR2082, Biomax.us), including 206 cases: 32 metastatic, 68 invasive ductal, 22 each of lobular and intraductal carcinomas, 4 each of squamous cell and lobular carcinoma in situ, 8 fibroadenoma, 16 each of hyperplasia and inflammation, 10 adjacent normal tissues, and 6 normal tissues. Staining protocols for all antibodies were established using the avidin-biotin complex staining procedure. Initial trials used the manufacturer’s suggested specimen preparation and staining conditions. Each protocol was then optimized for antigen retrieval, antibody dilution, and incubation conditions. A tissue known to be positive for the antigen of interest was used to titer the antibody and subsequently was stained with each investigative study. IHC staining of this array was performed using an anti-EphB2 antibody (Acris Antibodies Inc. #AM11063SU-N) diluted to 1/100, anti-ephrinB1 (R&D Systems #AF473), anti-ephrin-B2 (R&D Systems #AF496), anti-ephrin-B3 (R&D Systems #AF395), and anti-Ephrin-A5 antibody (R&D Systems #AF3743) diluted to 1/25. The antibodies were titrated to breast samples exclusively. Control TMA slides were negative controls that omitted the primary antibodies. Protein expression was measured semi quantitatively (0: absent, 1+: weak, 2+: moderate, and 3+: strong expression). The results were illustrated as the frequencies (%) of specimens with different staining levels. In addition to determining the staining intensity, the percentage of tumor cells within each and every sample that showed strong positive staining was recorded and used to generate a quantitative score, according to a modified histological score (H-Score) method [82,83]. Here, the intensity scores reflected the percentages of cells with the maximal intensity, and thus ranged between 0 and 300 (a score of 3 in 100% of cells). Since data were not normally distributed, instead of means we reported medians, along with the standard deviations. Consequently, we reported the interquartile range, which is the value at the first quartile (Q1) to the third quartile (Q3). Q2 is by definition the median. Therefore, the Q1 to Q3 range provides an idea of what the values are for 75% of the data. Each cancer progression category was compared to the “Non-Cancer” category used as a reference, using Mann–Whitney U tests. As per the manufacturer’s acknowledgment (biomax.us), “all tissue is collected under the highest ethical standards with the donor being informed completely and with their consent”, and “all human tissues are collected under HIPPA approved protocols”. The authors have no access to patient information.

### 4.3. Cell Culture

MCF10A cells are spontaneously immortalized non-transformed and non-tumorigenic human mammary epithelial cells [84]. They have been engineered to express Harvey-ras (H-ras), to derive a rare model able to precisely recapitulate the progressive alterations associated with the temporal development of human breast carcinomas [38]. The three-dimensional culture and immunostaining of MCF-10A mammary epithelial cells and derivatives was performed according to a published protocol [85]. Cells were cultured in DMEM/F-12 medium (Invitrogen, Waltham, MA, USA) supplemented with 5% horse serum (Invitrogen), 2 mM glutamine, 100 g/mL streptomycin, 100 IU/mL penicillin, 0.25 μg/mL ampicillin B, 100 ng/mL cholera toxin, 20 ng/mL EGF (Upstate Biotechnology, Lake Placid, NY, USA), 0.5 μg/mL hydrocortisone (Calbiochem, San Diego, CA, USA), and 10 μg/mL insulin.

### 4.4. Real-Time RT-PCR

Total RNA was isolated using the RNAquous-4 PCR Kit (Ambion, Austin, TX, USA) according to the manufacturer’s instructions. To perform real time quantitative RT-PCR, we followed the SYBR green protocol, using the iTaq Fast SYBR Green Supermix with ROX (Biorad, Hercules, CA, USA) as directed by the manufacturer. Primers were obtained from realtimeprimers.com (Elkins Park, PA, USA). All experiments were carried out in triplicates at least.

### 4.5. Cell Lysate Preparation and Immunoblotting

Cells were washed twice with phosphate buffered saline (PBS) and scraped in ice-cold lysis buffer (20 mM Hepes, pH 7.4, 1% NP40, 2 mM EDTA, 100 mM NaF, 10 mM pyrophosphate, 1 mM sodium vanadate) containing 1× protease inhibitor cocktail. Then, the cell lysates were solubilized by sonication and cleared by centrifugation at 14,000 rpm for 10 min at 4 °C. The primary antibodies used were anti-H-Ras (Calbiochem #OP23) and anti-beta-actin (Santa Cruz, Dallas, TX, USA, sc-47778), and the secondary was an HRP-conjugated secondary anti-mouse (Cell signaling, Danvers, MA, USA, 7076S).

### 4.6. Nomenclature

We follow the unified nomenclature for the Eph/ephrin family [86].

## Figures and Tables

**Table 1:** Survival outcomes derived from Kaplan–Meier analysis according to different clinico-pathological variables, EphB2 gene expression and different cognate ligand-encoding EFN genes. Abbreviations: RFS: relapse-free survival; OS: overall survival; DMFS: distant metastasis-free survival; HR: hazard ratio (confidence interval (CI) = 95%). The indicated *p*-values were calculated by the log-rank test. In bold, *p*-values of <0.05 are considered as statistically significant.

Variables			Number of Patients	EPHB2		EFNB1		EFNB2		EFNB3		EFNA5	
				HR	*p*-Value	HR	*p*-Value	HR	*p*-Value	HR	*p*-Value	HR	*p*-Value
**RFS**	All	All	3554	0.74	**2.1** **× 10^−6^**	0.77	**1.5** **× 10^−5^**	1.28	**3.5** **× 10^−5^**	0.9	0.091	0.72	**1.7** **× 10^−8^**
	Lymph Node Status	LN+	945	1.34	**0.011**	0.71	**0.0059**	1.29	**0.024**	0.83	0.15	1.09	0.43
		LN-	1813	1.16	0.1505	0.89	0.21	1.24	**0.026**	1.22	**0.03**	0.91	0.36
	Intrinsic Subtype	Basal	580	0.69	**0.0049**	0.62	**0.00022**	1.21	0.19	1.47	**0.0071**	0.68	**0.0036**
		Luminal A	1764	0.7	**0.00011**	0.76	**0.0027**	1.25	**0.019**	1.14	0.16	0.57	**3.6** **× 10^−9^**
		Luminal B	1002	0.71	**0.0034**	0.81	0.06	1.23	**0.048**	0.8	**0.031**	0.68	**0.00022**
		HER2+	208	0.5	**0.0018**	0.69	0.083	1.75	**0.0094**	1.37	0.14	0.67	0.062
**OS**	All	All	1117	1.2	0.14	0.75	**0.019**	1.26	0.065	0.83	0.14	0.84	0.23
	Lymph Node Status	LN+	197	1.71	**0.035**	0.78	0.33	0.7	0.22	1.47	0.14	1.67	0.059
		LN-	425	0.65	**0.044**	0.52	**0.0035**	1.51	0.073	0.72	0.14	0.6	**0.04**
	Intrinsic Subtype	Basal	204	0.47	**0.031**	0.63	0.1	0.75	0.31	2.05	**0.01**	0.69	0.2
		Luminal A	504	1.38	0.096	0.66	0.072	1.59	**0.031**	1.27	0.22	0.67	0.1
		Luminal B	320	1.48	0.082	0.62	**0.026**	1.41	0.19	0.7	0.097	1.49	0.074
		HER2+	89	0.67	0.31	1.65	0.2	1.57	0.24	1.65	0.31	2.37	0.1
**DMFS**	All	All	1609	1.51	**5.6** **× 10^−5^**	1.25	**0.038**	1.43	**0.00058**	1.21	0.061	0.86	0.19
	Lymph Node Status	LN+	337	2.68	**1.2** **× 10^−6^**	1.4	0.11	1.44	0.091	1.53	**0.046**	1.5	0.054
		LN-	896	1.46	**0.022**	1.32	0.07	1.39	**0.035**	1.32	0.063	0.86	0.3
	Intrinsic Subtype	Basal	219	1.52	0.14	0.71	0.19	1.34	0.29	1.67	0.058	1.29	0.42
		Luminal A	918	1.5	**0.0068**	1.36	0.058	1.55	**0.0061**	1.31	0.074	0.79	0.15
		Luminal B	361	2.15	**0.00016**	1.55	**0.024**	1.34	0.13	1.24	0.26	1.31	0.16
		HER2+	111	1.1424	0.14	1.77	0.082	4.58	**0.0055**	2.37	**0.017**	2.35	0.068

**Table 2:** A comparison of the median scores of different cancer progression categories within individual markers. Median (Q1–Q3), *** *p*-value < 0.0001. Intensity scores were calculated by multiplying the intensity score (3) by the percentage of cells with the maximal intensity, the maximal value being 300 (3 × 100%). Since data were not normally distributed, instead of means we report medians, along with the standard deviation. Consequently, we report the interquartile range, which range of the first quartile (Q1) to the third quartile (Q3). Q2 is by definition the median. Therefore, the Q1 to Q3 range provides an idea of what the values are for 75% of the data. ^#^ Includes normal tissue adjacent to a tumor, hyperplasia, mastitis, fibroadenoma, and normal tissue. * Ductal carcinoma metastases.

	EphB2	EphrinB1	EphrinB2	EphrinB3	EphrinA5
**Normal ^#^**	0 (0–0), reference	0 (0–0), reference	0 (0–0), reference	0 (0–0), reference	0 (0–0), reference
**DCIS**	80 (20–170), ***	5 (0–80), ***	45 (15–58), ***	10 (0–30), ***	0 (0–20), ***
**Ductal Invasive**	35 (0–120), ***	70 (30–140), ***	140 (30–180), ***	10 (0–90), ***	40 (10–140), ***
Metastasis *	90 (30–190), ***	65 (25–120), ***	160 (60–270), ***	35 (10–150), ***	50 (0–120), ***

## Data Availability

Data supporting reported results can be found at https://kmplot.com/analysis/.

## Abbreviations

EphB2	B-type Eph receptor 2
EFN	ephrin
RTK	receptor tyrosine kinases
TMA	tissue microarray
